# Landscape and Anthropogenic Factors Associated with Adult *Aedes aegypti* and *Aedes albopictus* in Small Cities in the Southern Great Plains

**DOI:** 10.3390/insects11100699

**Published:** 2020-10-13

**Authors:** Jordan D. Sanders, Justin L. Talley, Amy E. Frazier, Bruce H. Noden

**Affiliations:** 1 Department of Entomology and Plant Pathology, Oklahoma State University, Stillwater, OK 74078, USA; sdane@okstate.edu (J.D.S.); justin.talley@okstate.edu (J.L.T.); 2 Department of Geography, Oklahoma State University, Stillwater, OK 74078, USA; amy.frazier@asu.edu

**Keywords:** mosquito, *Aedes*, urban, Oklahoma, Great Plains

## Abstract

**Simple Summary:**

Mosquito-borne diseases are a growing human health concern in the United States. While recent studies have updated the distribution of *Aedes aegypti* in southern Great Plains, little is known about what factors can be used to predict where important mosquito species thrive in the region. The aim of the study assessed different factors associated with encountering adult container-breeding mosquitoes in small cities in southern Oklahoma. Collections using two types of traps were carried out over a ten week period from June to August 2017 along two geographical transects, each consisting of three cities, equally distant from the Red River/Texas border. After five rounds of collection, 6628 female mosquitoes were collected from 242 commercial or residential sites in six cities. Of the mosquitoes collected, 80% consisted of container-breeding species. Regionally, *Ae. aegypti* was most likely to be collected in cities closest to the Texas border while *Aedes albopictus* was spread throughout the region. In general, *Ae. aegypti* and *Ae. albopictus* were more associated with residential sites or sites featuring no or low vegetation. The study highlighted important factors involved in the distribution of *Ae. aegypti* and *Ae. albopictus* in small cities in the southern Great Plains.

**Abstract:**

As mosquito-borne diseases are a growing human health concern in the United States, the distribution and potential arbovirus risk from container-breeding *Aedes* mosquitoes is understudied in the southern Great Plains. The aim of the study was to assess landscape and anthropogenic factors associated with encountering adult container-breeding mosquitoes in small cities in southern Oklahoma. Collections were carried out over a 10 week period from June to August 2017 along two geographical transects, each consisting of three cities, equally distant from the Red River/Texas border. Mosquitoes were collected weekly using two trap types along with data for 13 landscape, vegetation, and anthropogenic variables. After five rounds of collection, 6628 female mosquitoes were collected over 2110 trap-nights involving 242 commercial or residential sites in six cities. Of the mosquitoes collected, 80% consisted of container-breeding species: *Aedes albopictus* (72%), *Culex pipiens* complex (16%) and *Aedes aegypti* (8%). Regionally, *Aedes aegypti* was more likely present in cities closest to the Texas border while *Ae. albopictus* was spread throughout the region. In general, *Ae. aegypti* and *Ae. albopictus* were significantly more present in sites featuring no or low vegetation and residential sites. Variables associated with *Ae. albopictus* presence and abundance varied between cities and highlighted the urban nature of the species. The study highlighted the distribution of *Ae. aegypti* geographically and within the urban context, indicated potential habitat preferences of container-breeding mosquito species in small towns, and demonstrated the usefulness of Gravid *Aedes* traps (GAT) traps for monitoring *Aedes* populations in urban habitats in small cities.

## 1. Introduction

Vector-borne diseases are a growing human health concern in the United States [1]. The emergence and repeated expansion of vector-borne human and animal diseases worldwide, and the increasing pace at which epidemics seem to occur, emphasize the continued need to surveille important disease vectors to prevent or respond to outbreaks. The recent rapid expansion of Chikungunya, Dengue, and Zika viruses through *Aedes* mosquitoes in Central and South America and the Caribbean region has focused attention on potential regions in the United States where epidemics could occur [2,3,4,5]. To mitigate potential outbreaks, studies have broadly mapped potential regions where two important vectors, *Aedes albopictus* (Skuse) and *Ae. aegypti* (L.), could potentially occur in the U.S. [6,7]. This renewed focus on surveillance and monitoring has provided critical resources to map mosquito communities and update distributions, which will aid mosquito control efforts in areas where it has been minimally applied.

Oklahoma is a unique state in which to envision potential arbovirus outbreaks in the southern Great Plains. An ecologically diverse state, the unique confluence of environmental patterns and small cities formed along rivers or other ecologically relevant areas make Oklahoma an ideal place in which to study ecological factors that could be involved in the spread of mosquito-borne disease [8]. While only *Culex*-transmitted West Nile Virus (WNV) is known to impact humans in the state [9], recent reports of Chikungunya, Dengue, and Zika have occurred in southern Texas, encouraging the need for surveillance and monitoring [1,10,11]. To date, our understanding of mosquito communities within Oklahoma remains rudimentary. Currently, 64 known species have been identified in the state [12,13], occurring in varying communities depending on ecoregion [14]. The recent re-establishment of *Ae. aegypti* [15] and discovery of *Ae. japonicus* [13] demonstrates the need for continued surveillance in this understudied region. 

Any successful control strategy begins by documenting where competent vector species reside and evaluates how mosquito communities differ among sites [9,16,17]. Given the critical lack of knowledge regarding the habitats in which container-breeding mosquitoes thrive in southern Oklahoma, the aim of the study was to create a baseline from which to study the ecology of container-breeding mosquitoes in small cities in the southern Great Plains. The objectives were to (1) determine the distribution of *Aedes aegypti* and *Aedes albopictus* in urban zones of different cities, and (2) identify landscape and anthropogenic variables related to their presence and abundance in central and western Oklahoma.

## 2. Materials and Methods

**Figure 1:**
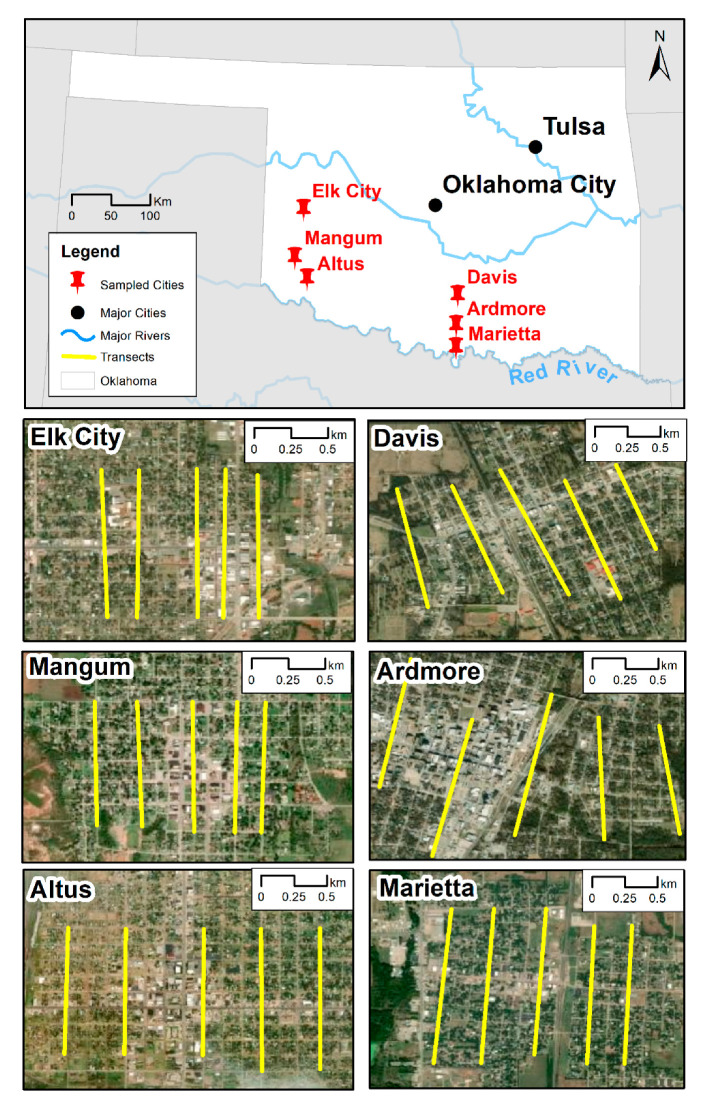
The six urban clusters in Oklahoma where mosquito collections were carried out between June and August 2017. The five transects in each city along which Gravid *Aedes* traps (GAT) were placed are provided.

**Study Area:** The study area consisted of two transects, each approximately 70 km in length, and each consisting of three ‘urban clusters’. According to the US Census Bureau, towns or cities with less than 50,000 residents are ‘urban clusters’ while those with more than 50,000 are ‘urban areas’ [18]. The southernmost cities in each transect were approximately equidistant from the Red River/Texas border (Figure 1). Mirroring reports of *Ae. aegypti* in northern Texas counties [7], the eastern transect consisted of three cities (Marietta, Ardmore, and Davis) along Interstate 35, traveling north from the Texas/Red River border in the central part of the state while the western transect consisted of three cities (Altus, Mangum, and Elk City) in the western part of the state (Figure 1). The two transects were selected based on the presence of *Ae. aegypti*-positive cities (Ardmore and Altus) [15] with similar sized populations that had urban cores large enough to place 30 mosquito traps and were similar distances from the Texas border [19]. In this study, the ‘urban core’ consists of the central part of the city with the highest densities of impervious surfaces and, normally, lower amounts of residential housing compared to surrounding areas.

Prior to choosing trapping sites, Oklahoma Cooperative Extension educators were contacted in each county where an urban cluster was chosen and permission meetings were conducted with city officials and local police to discuss the study objectives, identify site-specific methods of informing communities about the study, and confirm the safety of personnel at specific trap sites chosen within each urban area [19]. Upon approval from the mayor and chief of police, research personnel confirmed potential trapping sites in individual cities using Google Earth imaging based on vegetation and a visual determination of container availability. Once sites were selected, research personnel contacted each resident or industry to personally explain the study rationale and procedure. Verbal consent was provided by each resident or industrial property owner for mosquito trap placement in the front area of their property.

**Mosquito Sampling:** Two trapping methods were used to establish *Aedes* sp. distribution in each city. Gravid *Aede*s traps (GAT) (Biogents, Regensburg, Germany) were used to evaluate the distribution of *Aedes* species within urban cores while BG-Sentinel traps (Biogents, Regensburg, Germany) were used to randomly sample surrounding residential areas or area outside the city limits with the purpose of evaluating the extent to which various *Aedes* sp. were distributed in the areas outside of the urban core of a particular city. Sites within the core and outer city limits were labeled ‘residential’ (any type of housing) or ‘industrial’ (small businesses, town halls). ‘Rural’ sites consisted of homes on the outer limits of the city surrounded by open fields and not in a neighborhood. ‘Agricultural’ sites were located in areas of open fields such as crops with no homes or businesses. Using the U.S. Geological Survey (USGS) national land cover database (NLCD) developed by the Multi-Resolution Land Characteristics Consortium [20], the percentage of urban impervious surfaces and vegetation cover were considered during site selection. Due to container-breeding mosquito resting behavior, vegetation coverage around a site was assessed based on scale parameters described elsewhere in the methods.

Within each city, five 1000-m transects, located at least 200 m apart, were plotted using Google Earth to maximize mosquito population detection in the urban core [19,21] (Figure 1). Along each transect, four gravid *Aedes* traps (GAT) were placed approximately 250 m apart for a total of 20 traps/urban core area to ensure the collection of *Aedes* mosquitoes from that particular area [22,23]. Urban core transects in each city were surveyed every week (alternating transects each week) between June 12 and August 17, 2017. Each week, 60 GAT traps (20 per city) were placed on Monday and collected on Thursday (approximately 72 h of collection) establishing a total of 180 trap-nights per regional transect per week. GAT traps were placed in well-shaded areas at the front of the property to attract adult mosquitoes in the immediate vicinity. To maximize identification potential with quick mosquito knockdown, the inner surfaces of each GAT trap were treated with 10% permethrin (Durvet, Blue Springs, MO, USA). In addition, the GAT traps were placed on clear plastic plant saucers (Lowe’s, Mooresville, NC, USA) and submerged with water to inhibit ant infestations. 

To augment the urban core trapping, 20 additional sampling areas were identified for their ecological uniqueness (tree cover, clutter amounts) around the urban cores in each city using USGS NLCD data together with Google Earth. During the same GAT trap sampling period in each transect, 10 BG-Sentinel trap sites were randomly chosen, and traps were placed at each site for 20 h, beginning at noon until 8–9 am the next day [19]. The purpose of these traps was to determine adult host-seeking mosquito populations within the wider area around each urban core. 

**Mosquito Identification:** At the time of collection, mosquitoes were removed from the traps immediately with the use of microdissection forceps, placed into 7 dram vials, and stored in a Whynter 45-quart portable freezer (Whynter, Brea, CA, USA) at −20 °C prior to identification under a Labomed Luxeo 4Z dissecting microscope (Labomed Inc., Los Angeles, CA, USA). Using Darsie and Ward [24], each mosquito was identified to species unless unidentifiable due to damage. After identification, all mosquitoes were transferred to −20 °C freezers (Frigidaire, Dayton, OH, USA) until further processing. Due to southern Oklahoma being a hybrid zone, *Culex pipiens* and *Culex quinquefasciatus* were identified as *Culex pipiens* L. complex [25]. 

***Aedes aegypti* Confirmation Assay:** Molecular detection was utilized for several mosquitoes with identity markings rubbed off during collection. DNA was extracted from the legs of each mosquito using the manufacturer’s instructions (GeneJET, Genomic DNA Extraction Kit, Thermoscientific, Grand Island, NY, USA). The positive control was *Ae. aegypti* Liverpool strain continuously reared in the laboratory. The extracted mosquito DNA was tested using Polymerase Chain Reaction (PCR) primers and conditions that amplified a 361 bp region of the ND4 gene [26]: ND4-Forward primer (5′-ATTGCCTAAGG CTCATGTAG-3′) and ND4 Reverse (5′- TCGGCTTCCTAGTCGTTCAT- 3′) on a Bio-Rad C1000 Touch thermal cycler (BIO-RAD, Hercules, CA). Positive amplicons were sent to Oklahoma State University Core Facility to be bi-directionally sequenced. Resulting sequences were searched using the nucleotide BLAST database to determine the species of mosquito collected.

**GIS analysis:** Additional land cover variables were extracted from the 2011 NLCD tree canopy cover and developed imperviousness cartographic products, which are both derived from Landsat imagery at a spatial resolution of 30 m [27,28]. These site-specific variables included canopy cover at 100 m and 250 m and urban impervious surface at 100 m and 250 m. Buffers of 100 m and 250 m were created around each of the 242 site locations. The 250 m distance was chosen to reflect mosquito flight behavior of around 50–100 m, but for good measure, a 100 m buffer zone was set for a more accurate representation of mosquitoes in a given area [21]. The tree canopy cover and developed impervious surface rasters were clipped to each buffer, and the total % of each was aggregated within the buffer, resulting in four land cover variables per site: canopy cover at 100 m and 250 m, and urban impervious surface at 100 m and 250 m. USFS Oklahoma-based tree canopy cover and 2011 NLCD developed impervious surface layers and site point locations (Google Earth) were added in ArcMap to the explanatory variables (Excel) detailed below. 

**Field Data Collection Variables:** In addition to the landscape-based variables, additional site-specific variables were collected in the field including the numbers of visible containers, number of dogs, a measure of backyard clutter, and a visual assessment of percent vegetation (Table 1). 

‘Number of containers’ per site was calculated by visual assessment of the actual number of any item that could hold water such as flower vases, bird-baths, and old tires present in the front yards of homes or businesses. ‘Number of dogs’ around a house was also assessed visually during each visit with notes taken on how many canines were repeatedly present at each resident. ‘Backyard clutter’ was assessed using Google Earth imagery due to privacy limitations of inspecting backyards in person, with clutter categorized as low, medium, or high volume at each site or immediate surrounding area. ‘Low clutter’ was distinguished by mostly vegetation surrounding a site with one or two visible containers. ‘Medium clutter’ consisted of sites with 10+ containers. ‘High clutter’ sites had more than 25+ containers, such as an old car salvage yard or waste dump. ‘Percent vegetation’ followed the protocols of Walker et al. [29] and involved an estimation by visual examination by a single viewer for consistency. Sites were labeled in categories of one to four. A ‘Level 1′ (no_veg) site had 0–10% vegetation coverage, ‘Level 2′ (low_veg) had 10–25% vegetation coverage, ‘Level 3′ (med_veg) had 25–50% vegetation coverage, and ‘Level 4′ (high_veg) had 50–100% coverage. 

**Statistical Analysis:** The influence of regional and city-specific variables was tested on the presence/absence and mean abundance per trap-night of *Ae. albopictus*, *Ae. aegypti* and *Cx. pipiens*. Our first question focused on the influence of regional factors (transects, mean elevation, and latitude) on mean abundance per trap-night for all three species. Prior to analysis using SAS JMP Pro 15.0, mosquito species abundance values were log-transformed to improve the assumptions of normality and homogeneity of variance. Because of the significant differences by trap between the abundance of the three species, trap types were analyzed separately: egg laying mosquitoes (GAT traps) in urban core areas and host-seeking mosquitoes (Sentinel traps) in random sites around each city. Regional transect analysis included one-way ANOVA by transect as well as by trap type/transect. Tukey honest significant difference (HSD) was used to compare between cities after significant one-way ANOVA analyses. Logistic regression analysis was used to assess the influence of distance from Texas on species presence/absence. 

Since the presence of each species differed regionally and by city, our second question focused on the influence of city-specific factors on each mosquito species. Because of the low numbers of trapping events for *Ae. aegypti* and *Cx. pipiens* in many cities, initial analyses of all three species began with Pearson’s correlation (Fisher’s Exact if degrees of freedom = 1) (nominal variables) and linear regression (continuous variables) analyses of the influence of each landscape and anthropogenic variables on presence of each species (Table 1). Continuous variables were log-transformed (number of containers, number of dogs, Tree_100 and Tree_250) or square-root-transformed (Urban 100/Urban 250) to improve the assumptions of normality and homogeneity of variance. After initial analysis, we tested the influence of the variables by city in a stepwise logistic regression analysis with minimum Akaike information criterion (AICc) in a forward direction for only those cities in which there were sufficient numbers. Setting criteria for model inclusion at the 0.05 level, setup continued until the lowest Akaike information criterion (AICc) value was reached. AICc was used to correct for small sample sizes. The dependent variables were the presence or absence of each species in combined traps while independent variables included the all explanatory variables (Table 1).

Our third question focused on the influence of city-specific variables on the abundance of *Ae. albopictus* in the six cities. Initial analysis included one-way ANOVA (accompanied by Tukey HSD) of variables with log-transformed mean trap abundance of *Ae. albopictus*. Further analysis evaluated relationships between landscape, temporal and anthropogenic variables and abundance of *Ae. albopictus* in all cities using the generalized linear model (GLM) with log link and Poisson distribution. GLM analysis was not possible for *Ae. aegypti* and *Cx. pipiens* abundance due to low numbers and high variability between cities and traps. We fit separate models for each city/trap combination with *Ae. albopictus* abundance as the dependent variable and non-significant predictor variables were excluded in a stepwise manner for each model. We then selected the best model and validated the model using goodness of fit statistics involving deviance, overdispersion and prediction profiles. 

Lastly, we focused on the influence of the variables on mean trap abundance of each species. Analysis included one-way ANOVA analysis (accompanied by Tukey HSD) of variables on transformed mean trap abundance by trap type for each species in general then by city. The small numbers of *Cx. pipiens* collected in GAT traps (Table 2) meant that all *Cx. pipiens* analyses focused only on Sentinel trap results.

## 3. Results

### 3.1. Mosquito Collection

Between June and August 2017, 242 commercial or residential sampling sites were sampled in six cities along two regional bi-weekly transects in southern Oklahoma (Figure 1). A total of 6628 female mosquitoes were collected over 210 trap-nights/week producing a total of 900 trap deployments involving 2100 trap-nights during the summer of 2017 (Table 2). 

Of the mosquitoes collected, 80% consisted of container-breeding species: *Ae. albopictus* (72%) and *Ae. aegypti* (8%) with a relatively high number of *Culex pipiens c*omplex (16%)*. Aedes aegypti* was collected in all but one city (Table 2). Of the two types of traps used, BG-Sentinel traps captured 4621 (70%) of the mosquitoes while GAT traps captured 2007 (30%), despite doubling the GAT traps for each Sentinel trap in each city (Table S1). This was due, in part, to high production sites in Davis and Mangum captured by BG-Sentinel traps that produced 25–50% of *Ae. albopictus* and *Cx. pipiens* complex. Trapping rates for both trap types were relatively equal for *Ae. aegypti*. The highest number of *Ae. aegypti* were collected in the southernmost cities (Marietta and Altus) with one *Ae. aegypti* collected in Davis and six in Magnum. The majority of *Ae. albopictus* were collected in Mangum and Davis with fewer in the southernmost cities (Marietta and Altus) (Table 2). The trends in mosquito species abundance increased throughout the summer months with an increase of numbers after 1 July 2017 (Figure S1). 

**Figure 2:**
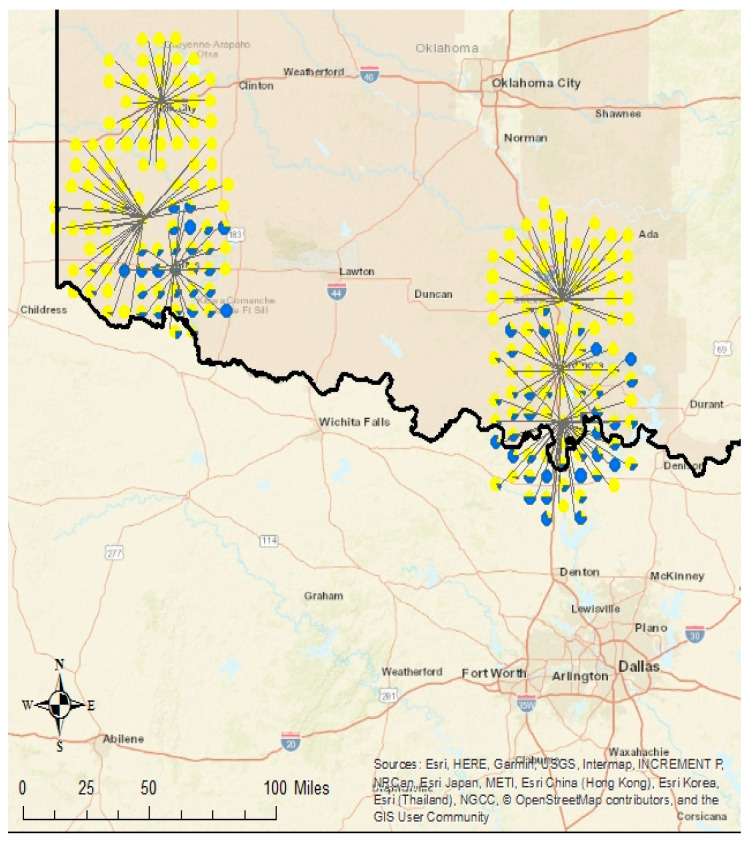
Proportion of total *Ae. aegypti* (blue) and *Ae. albopictus* (yellow) collected by site along the western and eastern Oklahoma transects between June and August 2017.

The overall proportion of *Ae. aegypti* (9%) to *Ae. albopictus* (91%) in the western transect was similar to the proportion of *Ae. aegypti* (11%) to *Ae. albopictus* (89%) in the eastern transect. However, the proportion of *Ae. aegypti* per city increased with proximity of the city to the Red River/Texas border (Figure 2). On the western transect, northernmost Elk City had zero *Ae. aegypti*/100% *Ae. albopictus,* while central Mangum had 2% *Ae. aegypti/*98% *Ae. albopictus* populations and southernmost Altus produced a ratio of 40% *Ae. aegypti*/60% *Ae. albopictus*. On the eastern transect, northernmost Davis had 0.1% *Ae. aegypti*/99.9% *Ae. albopictus*, while central Ardmore was 12% *Ae. aegypti*/88% *Ae. albopictus* and southernmost Marietta produced a ratio of 36% *Ae. aegypti*/64% *Ae. albopictus*. Other important species were collected as well in addition to container-breeders. The majority of *Aedes triseriatus* (87%) were collected in eastern transect cities (Davis and Ardmore) while very few *Cx. tarsalis* (*n* = 4) were collected in any city (Table 2). Interestingly, *Toxorhynchites rutilus* were collected in Sentinel traps in each eastern transect cluster but none in the western transect.

*Aedes aegypti* was mainly collected in three cities (Altus, Ardmore, and Marietta) (Table 2, Figure 2). In Altus, the southernmost city in the western transect, *Aedes aegypti* was collected in 75% of the traps (50% Sentinel, 100% GAT) while *Ae. albopictus* was collected in 72.5% of the traps (50% Sentinel, 95% GAT). Both species were collected in 67.5% (27/40) (8 Sentinel and 19 GAT) of the Altus traps. In Ardmore, the middle city in the eastern transect, *Aedes aegypti* was collected in 57.5% of the traps (65% Sentinel, 50% GAT) while *Ae. albopictus* was collected in 87.5% of the traps (80% Sentinel, 95% GAT). Both species were collected in 55% (22/40) (13 Sentinel and 9 GAT) of the Ardmore traps. In Marietta, the southernmost city in the eastern transect, *Aedes aegypti* was collected in 77.5% of the traps (60% Sentinel, 95% GAT) while *Ae. albopictus* was collected in 80% of the traps (65% Sentinel, 95% GAT). Both species were collected in 70% (28/40) (10 Sentinel and 18 GAT) of the Marietta traps.

### 3.2. Aedes aegypti Confirmation Assay

Six unknown mosquito samples from Davis and Mangum were tested by PCR for species identification. Five out of the six samples (one from Davis and four from Mangum) were confirmed using NCBI Blast with 100% sequence identity with known sequences of *Ae. aegypti* (KX580042.1; FJ428775.1) while the positive control had 100% sequence identity with a known sequence of *Ae. aegypti* Liverpool strain (MF194022.1). The remaining unknown sample had 100% sequence identity for known sequences of *Culex quinquefasciatus* or *pipiens* (GU188856.2; KX709954.1). 

### 3.3. Spatial Patterns of Mosquito Abundance

Assessing mean population abundance at a transect level, *Ae. albopictus* or *Ae. aegypti* abundance did not differ between eastern and western transects for any regional variables. In the eastern transect, mean *Ae. albopictus* abundance was significantly impacted by distance from Texas and higher latitude (F = 6.6267; *p* = 0.0016) with populations significantly higher in cities further from Texas (Davis and Ardmore) than in Marietta (Figure 1). While *Ae. aegypti* abundance did not differ by transect, significantly higher proportions of *Ae. aegypti* were collected closer to Texas in eastern (R^2^ = 0.26, df = 290, *p* ≤ 0.0001) (mean proportion/trap-night: Marietta = 0.42; Ardmore = 0.18; Davis = 0.002) and western (R^2^ = 0.32, df = 289, *p* ≤ 0.0001) (mean proportion/trap-night: Altus = 0.40; Mangum = 0.008; Elk City = 0.00) transects when compared with *Ae. albopictus*. This relationship was observed for both trap types used (eastern transect (GAT: R^2^ = 0.26, df = 298, *p* ≤ 0.0001; Sentinel: R^2^ = 0.24, df = 92, *p* ≤ 0.0001) and western transect (GAT: R^2^ = 0.32, df = 202, *p* ≤ 0.0001; Sentinel: R^2^ = 0.33, df = 87, *p* ≤ 0.0001). Identical results followed the pattern of lower latitudes for each transect. Mean *Cx. pipiens* abundance significantly differed by altitude between cities in Central Oklahoma (258–266 m) vs. Western Oklahoma (426–585 m) (F = 28.9916; *p* ≤ 0.0001). However, this relationship only occurred with host-seeking *Cx. pipiens* (Sentinel traps: (F = 19.2048; *p* ≤ 0.0001)). 

### 3.4. Site-Specific Assessment of Mosquito Presence and Abundance

#### 3.4.1. Aedes aegypti

Based on a total of 170 species-specific trapping events, presence of *Ae. aegypti* significantly differed between cities (Table 2) and week of sampling (mid-July into August) but did not differ by trap type. In general, *Ae. aegypti* were present at sites with no or low vegetation, residential sites in urban cores, and decreasing percentages of vegetation at 100 and 250 m from the trap site (Table S2). City-specific models developed by stepwise regression analysis reinforced these relationships, highlighting the significance of sampling period and lack of vegetation in addition to residential sites (Table 3). 

The mean trap-night abundance of *Ae. aegypti* significantly differed by trap type (F = 15.25; *p* = 0.0001) and sampling week (trending higher in July and August) (F = 4.78; *p* = 0.0014) but not by city (F = 2.99; *p* = 1.78). For GAT traps, city-specific bivariate analysis recorded significant abundance differences by sampling week (F = 6.12; *p* = 0.0006) and reduced numbers of containers in Altus (F = 5.79; *p* = 0.0215). For Sentinel traps, abundance was significantly higher in residential sites in Ardmore (F = 4.57; *p* = 0.0298).

#### 3.4.2. Aedes albopictus

Relatively large numbers of *Ae. albopictus* were collected in each city (Table 2) (total of 520 species-specific trapping events), allowing for full analysis of variables for presence and mean abundance for both trap types (Tables S3 and S4, Table 4 and Table 5). General univariate analysis of *Ae. albopictus* presence indicated significant spatiotemporal differences between sampling weeks and cities and city-specific association with decreasing vegetation together with significant relationships with anthropogenic variables involving increasing impervious surface, numbers of containers, presence of dogs, and residential sites (Table S3). Regression analysis of *Ae. albopictus* presence reinforced the importance of spatiotemporal differences between cities and sampling weeks with lower presence in traps in June in most cities (Table 4). City-specific differences highlighted association of *Ae. albopictus* presence with many variables including increasing percentage of impervious surface at 100 m and 250 m, decreasing tree cover at 100 and 250 m, medium vegetation, and increasing numbers of containers in urban clusters (Table 4).

Abundance of *Ae. albopictus* took on a different perspective, differing by trap type and cities (Table S4 and Table 5). For abundance of oviposition-seeking adult female *Ae. albopictus* (GAT traps), sampling week (particularly early July and early August) was significant in four of the six cities. *Aedes albopictus* abundance was significantly associated with high vegetation and increasing percentage of vegetation at 100 m and 250 m from the trap site, particularly in eastern transect cities. In regards to anthropogenic variables, GAT-trapped *Ae. albopictus* were associated with residential sites, medium amounts of clutter, and presence of dogs in different cities.

For abundance of adult host-seeking *Ae. albopictus* (Sentinel traps), sampling week was only important for one city. Abundance was significantly associated with increasing levels of vegetation, increasing levels of impervious surface at 100 m, increasing numbers of containers, except for Ardmore where the relationship was in the direction of decreasing impervious surface at 100 m (Table S3). Results from the GLM analysis reinforced these bivariate results (Table 5). Differing by city, GAT trap analysis emphasized the importance of sampling week, increasing percentages of vegetation and impervious surface at 100 m, industrial sites, and medium clutter. Abundance in Sentinel traps also varied by city with *Ae. albopictus* in central Oklahoma associated with rural sites, increasing impervious surface at 100 m and numbers of containers while *Ae. albopictus* in western Oklahoma were associated with sampling week, increasing vegetation at 100 m and 250 m, and increasing containers (Table 5). Of note, the model for adult host-seeking mosquitoes in Altus only became positive when vegetation was added to the other two variables, emphasizing the importance of vegetation in that hot, arid urban cluster.

#### 3.4.3. Culex pipiens Complex

Presence of *Cx. pipiens* also differed between cities (R^2^ = 0.0186; df = 5; *p* = 0.0204), sampling weeks (R^2^ = 0.0245; df = 4; *p* = 0.0026), and trap type (R^2^ = 0.1513; df = 1; *p* ≤ 0.0001). In general, the presence of *Cx. pipiens* was more likely at industrial (R^2^ = 0.0069; df = 1; *p* = 0.01790), agricultural (R^2^ = 0.0051; df = 1; *p* = 0.0391) or rural (R^2^ = 0.0097; df = 1; *p* = 0.0066) sites with limited vegetation (R^2^ = 0.0060; df = 1; *p* = 0.0266) or high amounts of clutter (R^2^ = 0.0059; df = 1; *p* = 0.0281). Due to limited numbers and variation between Sentinel trap sites, only low vegetation (R^2^ = 0.1241; df = 1; *p* = 0.0385) was significantly associated with *Cx. pipiens* presence in Elk City, OK, USA, and decreasing percentage of vegetation at 100 m (estimate (SE)= −6.63 + 3.28; ChiSq = 4.09; *p* = 0.0433) in Marietta. Abundance of *Cx. pipiens* differed significantly by trap type (F = 27.63; *p* = 0.0001) and city (majority collected in eastern transect cities) (F = 0.13; *p* = 0.0001) but not sampling week (F = 2.19; *p* = 0.0748) (Table S1). Mean *Cx. pipiens* abundance from Sentinel traps was associated with no vegetation (F = 6.73; *p* = 0.0203) in Marietta and high vegetation (F = 7.22; *p* = 0.0129) in Davis. 

## 4. Discussion

This study identified landscape, spatial, temporal and anthropogenic factors influencing the presence and abundance of adult female *Ae. aegypti* and *Ae. albopictus* in urban clusters in the southern Great Plains. The primary mosquito of interest was *Ae. aegypti*, a major transmitter of arboviruses to humans recently re-established in the northern Texas/southern Oklahoma region after not being detected for over 70 years [7,15,30,31]. Results indicated that (1) on a state-wide scale, *Ae. aegypti* is currently most prevalent in southern cities in the state, (2) GAT traps effectively target container-breeding *Aedes* species in urban settings but collect significantly fewer mosquitoes than BG-Sentinel traps and (3) unique factors in each small city indicate specific environments in which each container-breeding mosquito species thrive. The variables analyzed were collected from six urban clusters in southern Oklahoma that have relatively small populations (~2500+ people) [32]. These small cities are called ‘urban’ because impervious surface is present in higher density than the surrounding areas, but they may still be considered ‘rural’ due to size of human populations and abundance of vegetation throughout the communities. Landscape and anthropogenic variable analyses identified a few characteristics that impact container-breeding mosquito species across the region, but, overall, variables differed in each urban cluster in relation to the presence and abundance of oviposition-seeking and host-seeking adult container-breeding mosquitoes. 

*Aedes aegypti* in southern Oklahoma is significantly linked with distance of the urban cluster from Texas. The re-discovery of established populations of *Ae. aegypti* in Oklahoma occurred only in 2016 when mosquito surveillance activities began to focus on the southwestern regions, a previously un-monitored region of the state [12,15]. Since then, *Aedes aegypti* has been reported in most Texas counties bordering the southern and western borders of Oklahoma [7] so it was not surprising to find *Ae. aegypti* more established in the southern most cities. The limited distribution of *Ae. aegypti* is notable compared with *Ae. albopictus,* which is spread throughout all the small cities. While *Ae. aegypti* is firmly established along the Texas/Oklahoma border [7,14,15], results from this study indicate it may be spreading northward. On the west side, *Ae. aegypti* is expanding with the collection of four females in Mangum and subsequent collection of one female in Hobart (south-central Oklahoma) (Thomas Hess, unpublished data). The detection of one female in Davis, central Oklahoma, was interesting but we suspect it was from a temporary population as no others were collected at that site in 2017 or in several attempts in 2018. Geographically separated from established populations in central Oklahoma by the Arbuckle mountains (natural barriers of dispersal [33]), the movement from *Ae. aegypti*-infested area below the mountain range to an urban area above the range most likely occurred via human-aided vehicle dispersal [34,35]. This needs further follow-up as the potential northern expansion of *Ae. aegypti* could further increase the risk for an arbovirus epidemic to Oklahoma City, OK, USA, the largest metropolitan area in the state, 97 km north of Davis, OK, USA.

One of the most interesting results from the study involved potential interactions between *Ae. aegypti* and *Ae. albopictus*. In the three cities where most *Ae. aegypti* were collected, 55–70% of the traps also collected *Ae. albopictus*. This indicated that both species are living within the same habitats, particularly in the urban core areas in cities closest to Texas where 90–95% of the traps collected both species (Table 2). The presence of both egg-laying and host-seeking adult female *Ae. albopictus* and *Ae. aegypti* was significantly more likely in areas of lower vegetation, particularly in southernmost urban clusters with the highest presence of *Ae. aegypti*. These areas of 0–25% vegetation were also significantly more likely to have increasing numbers of containers and have medium to high clutter. The association of higher presence with 0–25% vegetation surrounding a trap may also indicate that higher vegetation amounts may obstruct trap odors or visual cues from females looking to oviposit or to rest [36,37]. These areas of low vegetation also correlate with an increase in impervious surface in urban cores, a predictive habitat of *Ae. aegypti* reported by others [21,38,39]. In Altus, OK, USA, the presence of *Ae. aegypti* was more likely in low vegetation and residential sites while *Ae. albopictus* was associated with lower percentage of vegetation. These relationships with vegetation may be appropriate given the general vegetative layout of Altus in dry, arid south western Oklahoma. Both species were collected in almost equal numbers of traps throughout the city, most notably the majority of the GAT traps in the urban core area, an area of considerably limited vegetation which may have biased the model. In Marietta, OK, USA, the southernmost city in the eastern transect, a similar presence of both species was recorded with the majority of both collected in GAT traps in the urban core. The lack of association of presence of any mosquito species with vegetation variables in Marietta, OK, USA, suggests that vegetation likely plays a limited role in the habitat selection. However, that relationship changed in Ardmore, OK, USA, with the presence of both species significantly associated with lower percentages of vegetation. Interestingly, *Aedes albopictus* (87% of traps) was considerably more present throughout Ardmore, OK, USA, than *Ae. aegypti* (58% of traps) with almost all GAT traps collecting *Ae. albopictus* compared with only half collecting *Ae. aegypti*. While most Ardmore, OK, USA, neighborhoods surrounding the urban core have a high proportion of trees, the urban core is surprisingly low in vegetation which may have factored into the model.

All three species of mosquitoes were significantly more likely to be present and most abundant between mid-July and end of sampling in August. This association with later sampling times is most likely directly related to higher temperature and relative humidity as reported for all three species in similar settings [40,41]. In US-based studies, *Ae. aegypti* has been associated with hotter, more arid urban zones ((South Florida [21,40] and the Texas–Mexican border [41]), which is a probable reason for increased presence and abundance in southwestern Oklahoma cities, which are hotter and drier than northern Oklahoma cities. Like *Ae. aegypti*, *Ae. albopictus* was significantly impacted by temporal factors as evidenced by significant differences between sampling week. The impact of abiotic and biotic factors on the development of *Ae. albopictus* in urban zones is well studied [42,43,44,45] and appears to be similar in the southern Great Plains. One interesting result was the higher abundance of *Cx. pipiens* in the eastern transect, a regional preference noted by an earlier study [12]. Future studies are needed to identify how climatic components limit *Cx. pipiens* development and expansion into western Oklahoma where *Cx. tarsalis* is the major *Culex* species [12]. 

The ubiquity of *Ae. albopictus* throughout all small cities sampled was characterized by the unique city-specific variables that characterized the presence and abundance of the species. The presence of *Ae. albopictus* throughout a given urban cluster was significantly associated with increasing urbanization (levels of impervious surface and numbers of containers) and decreasing levels of vegetation (Table 4), much like that of *Ae. aegypti*. Including data from both trap types, the association of *Ae. albopictus* presence with urbanization in these small cities may indicate different habitat structure compared with studies in highly urbanized environments [43,46]. Variables associated with *Ae. albopictus* abundance varied considerably between urban clusters (Table 5). The mean trap-night abundance of *Aedes albopictus* collected using GAT traps in urban core areas was associated with decreasing levels of impervious surface in Elk City, OK, USA, a dry, arid city in western Oklahoma, but with increasing tree cover in Ardmore, OK, USA, and Mangum, OK, USA, two cities with relatively high amounts of tree cover. Higher numbers of GAT-trapped *Ae albopictus* in Davis, OK, USA, and Altus, OK, USA, two urban clusters with very different environmental conditions, were associated with industrial areas, which were also associated with low levels of vegetation. On the other hand, abundance of *Ae. albopictus* changed with host-seeking Sentinel traps spread throughout a given community (Table 5). Higher abundance of host-seeking *Ae. albopictus* was associated with decreasing levels of urbanization (decreasing percentage of impervious surface, numbers of containers) and increasing levels of vegetation (increasing percentage of tree cover, rural sites). While biases in trap placement may have led to some of these ambiguous results, overall, it aligns with other landscape-focused studies in the United States [45,46,47,48]. In general, *Ae. albopictus* is non-discriminate in its use of habitat, continuously displaying a plasticity that allows it to adapt easily and thrive in any combination of vegetation and urban condition. The low R^2^ values indicate the land cover variables do not fully explain what is really driving the presence and abundance at a biologically relevant level in the different cities. The presence of *Ae. albopictus* in the southern Great Plains since the late 1990s [49] suggests that the species has had ample time to adapt to whatever conditions may occur within the specific microclimates and habitats of the small cities. 

While not the main objective of the study, results confirmed that GAT traps are an excellent low-cost alternative for determining the distribution of container-breeding *Aedes* species in small cities. GAT traps were developed as means to collect adult gravid female container-breeding *Aedes* species [36] and were tested in field-conditions involving large urban areas [50]. BG-Sentinel traps, alternatively, were designed for host-seeking mosquitoes [51]. In general, GAT traps effectively trapped adult container-breeding *Aedes* species in all of the urban clusters as the presence of *Ae. aegypti* and *Ae. albopictus* did not significantly differ by trap types. However, as reported by others, Sentinel traps collected significantly more *Ae. albopictus* than GAT traps [37,50,52], while *Ae. aegypti* abundance did not differ by trap type [52]. The collection of *Ae. aegypti* in 95–100% of the GAT traps in the two most southern urban clusters potentially indicates an advanced level of invasion versus collection in 50% of GAT traps in Ardmore which is further north. This invasion of *Ae. aegypti* into Ardmore has probably occurred within the last 10 years, when the last mosquito surveillance was carried out [12].

While all attempts were made to adjust for limitations, it is difficult to account for everything in a geographically-varied and labor-intensive study. While a complete season of sampling (until end of October) may have provided a more complete picture of how vegetation and other variables impact container-breeding communities, collections were terminated before populations of *Ae. aegypti* and *Ae. albopictus* normally begin to increase in Oklahoma due to funding cessation beyond our control. The period of sampling, however, did provide adequate amount of time to monitor the rise of population numbers in the season and indicated how different variables impact species presence and abundance. As one of the first to evaluate landscape variables with container-breeding mosquito species collected using GAT traps in small cities, the results may have been biased for the urban contexts in which the study was carried out. However, the widespread detection of the presence of container-breeding species, especially *Ae. aegypti*, by GAT traps demonstrated the importance of these traps in urban mosquito surveillance programs. Additionally, the use of permethrin in the GAT traps may have been repellant at some stages of the project. As a comparison was not done between impregnated and non-impregnated traps when first initiated, it may have limited collection numbers in some places. In regard to various variables, restriction of collections to areas in the front of residences and businesses may have limited the actual numbers of mosquitoes collected and as well as reduced the actual numbers of containers counted at a particular site. Conversely, establishing clutter levels using Google Earth images, which may not be up-to-date, may also have produced some variable bias. 

## 5. Conclusions

In conclusion, this study identified unique relationships of container-breeding mosquitoes with landscape and anthropogenic variables in an understudied region of the US. In addition to confirming the presence of established populations in two new counties (Jackson county (Altus, OK, USA) and Love county (Marietta, OK, USA)), *Aedes albopictus* is now reported in 72 of the 77 counties of Oklahoma [14,49]. The association of *Ae. aegypti* and *Ae. albopictus* populations in urban landscapes has mainly been studied in large urban centers in the US [21,35,40,41,43,45,46,47,48,53]. The unique landscapes of small urban clusters throughout the southern Great Plains where *Aedes* species thrive provide challenges for conceptualizing regional mosquito management. In considering how to effectively control container-breeding species, it is apparent that specialized management plans need to be developed for each city with consideration for vegetation and different anthropogenic variables. While providing a baseline on which to begin developing effective mosquito control management strategies, more research, specifically into variables involved with the ecology and distribution of *Ae. aegypti*, are needed to effectively mitigate future arboviral outbreaks in the region. 

## Figures and Tables

**Table 1:** Explanatory variables collected during summer 2017 for habitat preference analysis.

Explanatory Variables	Descriptor
#_containers	Number of containers in visibility from site location
# dogs	Number of visible canines around site location
dog presence/absence	Presence/absence of visible resident canine(s) around site
clutter density	Low, medium, high
percent vegetation	No_veg (level 1), Low_Veg (level 2), Med_veg (level 3), High_veg (level 4)
Urban_100	Total amount of impervious surface within 100 m of the site
Urban_250	Total amount of impervious surface within 250 m of the site
Tree_100	Total amount of tree canopy cover within 100 m of the site
Tree_250	Total amount of tree canopy cover within 250 m of the site
Week	Sampling period
Residential	Site location in a neighborhood
Industrial	Site location at a business or industrial area
Agricultural	Site location in an agricultural setting
Rural Sites	Site location in the outer limits surrounded by open fields, not in neighborhood

**Table 2:** Mosquito species collected in six Oklahoma cities using two trapping methods between June and August 2017.

Species	City						
	Eastern Transect			Western Transect			
	Marietta	Ardmore	Davis	Altus	Mangum	Elk City	Total
*Aedes aegypti*	197	90	1	253	4	0	547
*Ae. albopictus*	345	649	1266	384	1715	432	4791
*Ae. epactius*	9	16	35	35	13	11	119
*Ae. sollicitans*	0	0	0	0	0	3	3
*Ae. triseriatus*	0	23	25	1	0	6	55
*Ae. vexans*	0	0	0	1	0	0	1
*Anopheles pseudopunctipennis*	0	0	0	0	0	1	1
*An. punctipennis*	0	0	2	0	2	2	6
*An. quadrimaculatus*	0	0	0	0	1	1	2
*Culex erraticus*	2	0	9	1	1	2	15
*Cx. nigripalpus*	0	0	0	1	0	0	1
*Cx. pipiens* (complex)	264	108	562	50	40	21	1045
*Cx. tarsalis*	0	0	0	0	3	1	4
*Cx. territans*	0	0	3	0	0	4	7
*Psorophora ciliata*	0	0	0	0	0	2	2
*Ps. cyanescens*	0	0	1	4	1	9	15
*Ps. ferox*	0	1	0	0	0	0	1
*Toxorhynchites* *. rutilus*	3	5	5	0	0	0	13
Total	820	892	1909	730	1782	495	6628

**Table 3:** Stepwise logistic regression analysis of *Ae. aegypti* with minimum Akaike information criterion (AICc) in a forward direction with habitat-related variables between June and August 2017.

	Independent Variable	Estimate	Std Error	ChiSq	CL	*p*
	Eastern transect					
**Ardmore**	Intercept	1.6940914	0.322488	27.6	.	<0.0001 *
R^2^ = 0.1983	Weeks 1 and 2 vs. others	1.1771011	0.324021	13.2	0.61–1.92	0.0003 *
	Low Vegetation	0.7154231	0.220261	10.55	0.29–1.16	0.0012 *
**Marietta**						
R^2^ = 0.1900	Intercept	0.73964512	0.2161211	11.71	.	0.0006 *
	Weeks 1 and 2 vs. others	1.20626465	0.2161211	31.15	0.81–1.66	<0.0001 *
	**Western transect**					
**Altus**	Intercept	0.8293264	0.273737	9.18	.	0.0024 *
R^2^ = 0.2599	Weeks 1 and 2 vs. others	1.3700909	0.242377	31.95	0.92–1.88	<0.0001 *
	Residential sites	0.5367054	0.231508	5.37	0.093–1.01	0.0204 *
	No Vegetation	0.489398	0.226246	4.68	0.06–0.95	0.0305 *

* *p* ≤ 0.05 significant.

**Table 4:** Stepwise logistic regression analysis of *Ae. albopictus* presence with minimum AICc in a forward direction with landscape, spatiotemporal and anthropogenic variables in six cities in Oklahoma between June and August 2017.

	Eastern Transect						Western Transect				
	Independent variable	Estimate	Std Error	CI (95%)	*p*		Independent variable	Estimate	Std Error	CI (95%)	*p*
**Davis**						**Elk City**					
R^2^ = 0.0936	Intercept	−1.2978944	0.5758527	.	0.0242 *	R^2^ = 0.1003	Intercept	−1.3910586	0.501634	.	0.0056 *
	Week 2 vs. others	0.79691911	0.2202347	0.37–1.24	0.0003 *		Weeks 1 and 2 vs. others	0.51198048	0.1800807	0.16–0.87	0.0045 *
	Urban_250	4.14610868	2.0561731	0.22–8.33	0.0438 *		Urban_100	3.44103666	1.5014318	0.56–6.48	0.0219 *
							Med_Veg	0.63290445	0.2090189	0.24–1.06	0.0025 *
**Ardmore**						**Mangum**					
R^2^ = 0.0826	Intercept	0.82541703	0.3351642	.	0.0138 *	R^2^ = 0.2351	Intercept	−0.8020088	0.2138956	.	0.0002 *
	Weeks 2 and 5 vs. others	0.75887535	0.2294639	0.33–1.24	0.0009 *		Weeks 1 and 2 vs. others	1.27743253	0.2138956	0.88–1.72	<0.0001 *
	Tree_100	−1.4247312	0.6611617	−2.75–−0.15	0.0312 *						
**Marietta**						**Altus**					
R^2^ = 0.2792	Intercept	−1.5599924	0.7086991	.	0.0277 *	R^2^ = 0.0828	Intercept	0.84384565	0.3757964	.	0.0247 *
	Weeks 1 and 2 vs. others	1.15226096	0.3362427	0.53–1.87	0.0006 *		Week 1 and 2 vs. others	0.62962077	0.1793985	0.28–0.99	0.0004 *
	Dog_Presence	−0.7307708	0.2656929	−1.27–−0.22	0.0060 *		Tree 250	−3.0729878	1.4726262	−6.04–−0.23	0.0369 *
	Num_Containers	1.10144786	0.299501	0.56–1.74	0.0002 *						

* = significant result.

**Table 5:** Generalized linear model (GLM) analysis of mean abundance of *Ae. albopictus* by trap-night with landscape, spatiotemporal and anthropogenic variables between June and August 2017.

**GAT Traps**											
	**Eastern Transect**						**Western Transect**				
	**Independent variable**	**Estimate**	**Std Error**	**CI (95%)**	***p***		**Independent Variable**	**Estimate**	**Std Error**	**CI (95%)**	***p***
**Davis**	Intercept	−0.392052	0.2283544	−0.902, 0.017	0.0183 *	**Elk City**	Intercept	0.8746491	0.3687235	0.14, 1.60	0.0192 *
	Week 1	−1.075915	0.3761278	−1.94, −0.42	0.0003 *		Urban_100	−3.664891	1.3454824	−6.44, −1.10	0.0034 *
	Week 2	0.3292866	0.21952	−0.12, 0.75	0.1466						
	Week 3	0.2616456	0.2057748	−0.16, 0.66	0.2058						
	Week 4	0.5852899	0.1690119	0.25, 0.93	0.0003 *						
	Med Clutter	−0.274869	0.0949419	−0.46, −0.08	0.0055 *						
	Industrial	0.4647822	0.2129124	0.08, 0.95	0.0076 *						
**Ardmore**	Intercept	−0.692742	0.2574502	−1.32, −0.24	0.0001 *	**Mangum**	Intercept	−0.940648	0.2611862	−1.47, −0.43	<0.0001 *
	Week 1	−1.794448	0.7779615	−3.99, −0.62	<0.0001 *		Week 1	−0.925338	0.3232383	0.0, −0.35	0.0004 *
	Week 2	0.2019992	0.3152251	−0.42, 0.89	0.5933		Week 2	−0.466729	0.2444934	−0.98, −0.02	0.0470 *
	Week 3	0.8659301	0.2364756	0.45, 0.0	<0.0001 *		Week 3	1.0037865	0.1340088	0.75, 1.28	<0.0001 *
	Week 4	0.8187788	0.246799	0.38, 1.42	<0.0001 *		Week 4	0.121549	0.1655347	−0.21, 0.45	0.4094
	Tree_100	0.7141316	0.3545683	0.01, 0.0	0.0479 *		Tree_100	2.1172071	0.5942898	0.94, 3.31	0.0004 *
**Marietta**	Intercept	−0.237518	0.1406443	−0.53, 0.02	0.0289 *	**Altus**	Intercept	−1.220092	0.4427689	−2.40, −0.50	<0.0001 *
	Week 1	−0.120377	0.3841283	−0.99, 0.55	0.6942		Week 1	−0.223027	0.2880166	−0.83, 0.32	1
	Week 2	−0.048433	0.2632948	−0.60, 0.44	1.0000		Week 2	−0.290156	0.3293038	−1.00, 0.31	0.5914
	Week 3	0.4270776	0.2148863	0.0, 0.84	0.0415 *		Week 3	1.1361697	0.1737104	0.81, 1.50	<0.0001 *
	Week 4	0.3346308	0.2207547	−0.11, 0.76	0.1168		Week 4	−1.217254	0.4490468	0	0.0004 *
							Industrial	0.6660607	0.4247139	0.0, 1.82	0.0081 *
**Sentinel Traps**											
	**Eastern Transect**						**Western Transect**				
	**Independent variable**	**Estimate**	**Std Error**	**CI (95%)**	***p***		**Independent variable**	**Estimate**	**Std Error**	**CI (95%)**	***p***
**Davis**	Intercept	0.4772758	0.1650725	0.12, 0.78	0.0190 *	**Elk City**	Intercept	−1.194775	0.4853312	0.0, −0.30	0.0033 *
	Rural sites	0.3347099	0.1650725	0.03, 0.69	0.0212 *		# Containers	0.390101	0.1235389	0	0.0012 *
							Tree_250	3.2080804	1.0549301	0	0.0009 *
**Ardmore**	Intercept	1.1541507	0.2134228	0.73, 1.57	<0.0001 *	**Mangum**	Intercept	0.5314689	0.1603245	0.19, 0.82	0.0192 *
	Urban_100	−1.656097	0.7662041	−3.23, 0.0	0.0194 *		Week 1	0.0062933	0.2959498	−0.62, 0.56	1
							Week 2	0.1366674	0.4018702	−0.77, 0.84	1
							Week 3	0.5005273	0.2160474	0.08, 0.93	0.0131 *
							Week 4	−1.174995	0.411993	−2.12, −0.46	0.0006 *
**Marietta**	Intercept	1.2546588	0.3241852	0.54, 1.83	0.0028 *	**Altus**	Intercept	−0.582725	0.3565931	−1.36, 0.07	0.0337 *
	# Containers	−0.555369	0.1793199	−0.92, −0.20	0.0031 *		Tree_100	4.8654761	1.6249095	1.83, 8.38	0.0007 *
							Dog_Pres	0.452842	0.2334301	0.0, 0.98	0.0241 *
							No Veg	−0.414189	0.1757211	−0.76, −0.06	0.0241 *

* = significant result (*p* ≤ 0.05).

## Supplementary Materials

The following are available online at https://www.mdpi.com/2075-4450/11/10/699/s1, Figure S1: mean abundance of three mosquito species in six Oklahoma cities by sampling week between June and August 2017. Table S1: mosquito species collected in six Oklahoma cities using (a) BG gravid *Aedes* traps (GAT) and (b) BG-Sentinel traps between June and August 2017. Table S2: univariate analysis of *Aedes aegypti* presence by habitat-related variables collected in three cities in Oklahoma between June and August 2017. Results presented are significant (*) and close to significant results for purposes of pattern recognition. Table S3: univariate analysis of *Aedes albopictus* presence by habitat-related variables collected in six cities in Oklahoma between June and August 2017. Results presented are significant (*) and close to significant results for purposes of pattern recognition. Table S4: univariate analysis of *Aedes albopictus* abundance by habitat-related variables by two different traps in six cities in Oklahoma between June and August 2017. Results presented are significant (*) or close to significant results for purposes of pattern recognition.
