# Supplementary material for: Landscape and Anthropogenic Factors Associated with Adult Aedes aegypti and Aedes albopictus in Small Cities in the Southern Great Plains
Source: Insects. 2020 Oct 13;11(10):699. doi: 10.3390/insects11100699 (PMC7602065; doi:10.3390/insects11100699)
Supplement: Supplementary file 1 [file insects-11-00699-s001.pdf]

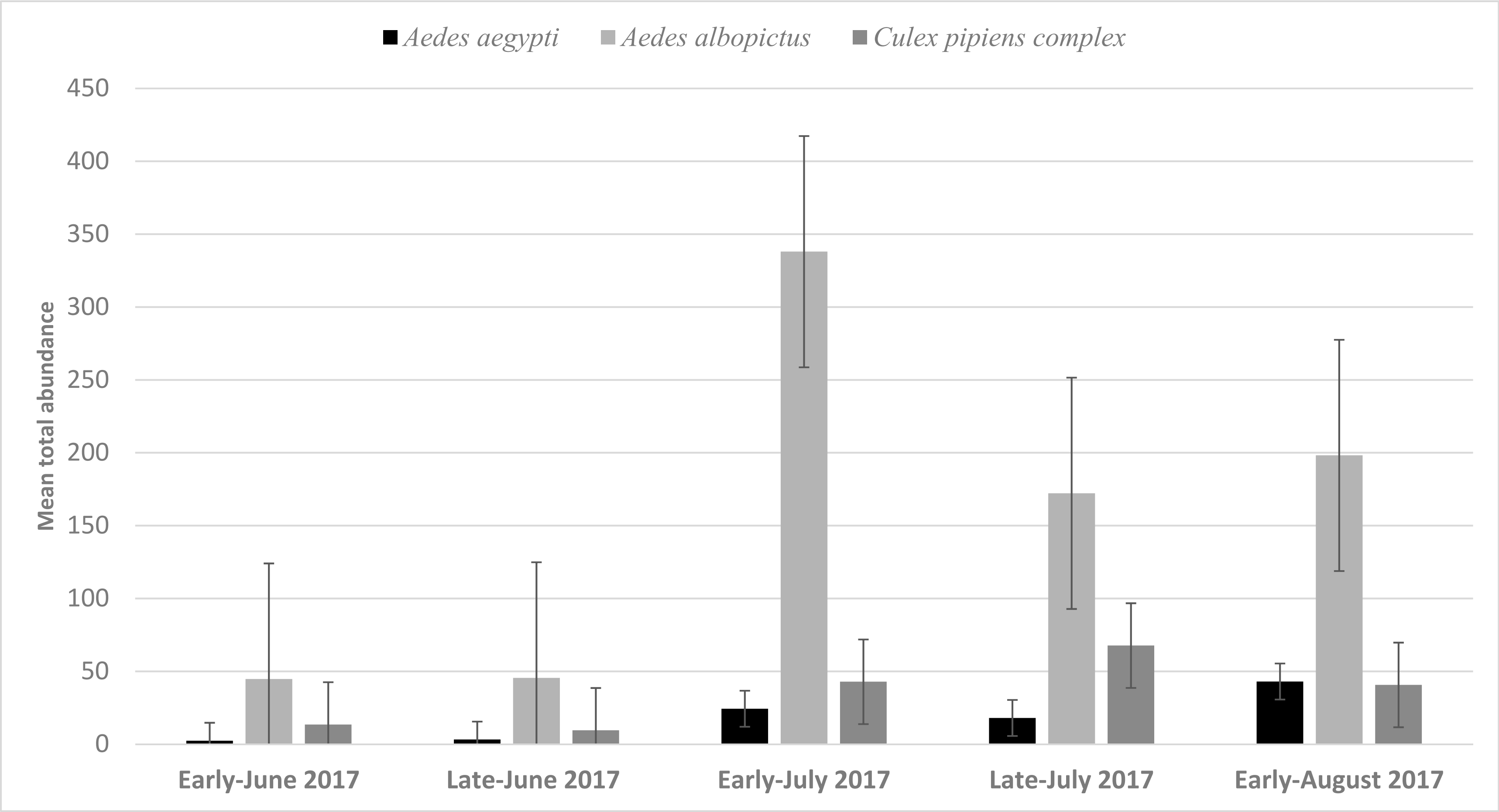

Figure S1. Mean abundance of three mosquito species in 6 Oklahoma cities by sampling week between June and August 2017.

Table S1. Mosquito species collected in six Oklahoma cities using a) BG gravid *Aedes* traps (GAT) and b) BG-Sentinel traps between June and August 2017.

**a. GAT**							
Species	Eastern transect			Western transect			
	Altus	Ardmore	Davis	Elk City	Mangum	Marietta	Total
Ae. aegypti	125	22	0	0	2	109	258
Ae. albopictus	200	219	353	218	456	197	1643
Ae. epactius	23	0	0	3	3	0	29
Ae. sollicitans	0	0	1	0	0	0	1
Ae. triseriatus	0	16	7	1	0	0	24
Ae. vexans	0	0	0	0	0	0	0
An. pseudopunctipennis	0	0	0	0	0	0	0
An. puncticpennis	0	0	1	0	0	0	1
An. quadrimaculatus	0	0	0	0	0	0	0
Cx. erraticus	1	0	1	0	0	0	2
Cx. nigripalpus	0	0	0	0	0	0	0
Cx. pipiens complex	12	6	4	6	4	1	33
Cx. tarsalis	0	0	0	0	0	0	0
Cx. territans	0	2	3	0	0	0	5
Ps. ciliata	0	0	0	0	0	0	0
Ps. cyanescens	1	0	0	9	1	0	11
Ps. ferox	0	0	0	0	0	0	0
Tx. rutilus	0	0	0	0	0	0	0

**b. BG Sentinel**								
**Species**	Eastern transect	Western transect						
		Altus	Ardmore	Davis	Elk City	Mangum	Marietta	Total
Ae. aegypti	128	68	1	0	4	88	289	
Ae. albopictus	184	430	913	214	1259	148	3148	
Ae. epactius	12	16	35	8	10	9	90	
Ae. sollicitans	0	0	2	0	0	0	2	
Ae. triseriatus	1	7	18	5	0	0	31	
Ae. vexans	1	0	0	0	0	0	1	
An. pseudopunctipennis	0	0	0	1	0	0	1	
An. puncticpennis	0	0	1	2	2	0	5	
An. quadrimaculatus	0	0	0	1	1	0	2	
Cx. erraticus	0	0	8	2	1	2	13	
Cx. nigripalpus	1	0	0	0	0	0	1	

Cx. pipiens complex	38	102	558	15	36	263	1012
Cx. tarsalis	0	0	0	1	3	0	4
Cx. territans	0	2	0	0	0	0	2
Ps. ciliata	0	0	2	0	0	0	2
Ps. cyanescens	3	0	1	0	0	0	4
Ps. ferox	0	1	0	0	0	0	1
Tx. rutilus	0	5	5	0	0	3	13

Table S2. Univariate analysis of *Aedes aegypti* presence by habitat-related variables collected in three cities in Oklahoma between June and August 2017. Results presented are significant (*) and close to significant results for purposes of pattern recognition.

	**Eastern transect**						**Western transect**		
	**Ardmore**			**Marietta**			**Altus**		
	R^2	DF	P	R^2	df	P	R^2	DF	P
Sampling Week	0.1743	4	0.0002*	0.2003	4	<0.0001*	0.2284	4	<0.0001*
No vegetation			NS			NS	0.0174	1	0.0436*
Low vegetation	0.0720	1	0.0007*			NS			NS
Residential sites	0.0341	1	0.0159*			NS	0.0208	1	0.0311*
Urban (vs Rural)			NS	0.0190	1	0.0404*	0.0196	1	0.0514
	Estimate (SE)	ChiSq	P	Estimate (SE)	ChiSq	P	Estimate (SE)	ChiSq	P
Tree 100			NS	-4.14+1.94	4.53	0.0333*	-2.07±1.19	3.02	0.0822
Tree 250			NS			NS	-2.44+1.42	2.96	0.0851

* = significant (Pearson (DF>1/Fisher's Exact (DF=1).

Table S3. Univariate analysis of *Aedes albopictus* presence by habitat-related variables collected in 6 cities in Oklahoma between June and August 2017. Results presented are significant (*) and close to significant results for purposes of pattern recognition.

**Eastern transect**	**Davis**			**Ardmore**			**Marietta**		
	R^2	DF	P	R^2	DF	P	R^2	DF	P
Sampling Week	0.1198	4	0.0002*	0.1035	4	0.0003*	0.0837	4	0.0015*
Residential sites			NS	0.0194	1	0.0442*			NS
	Estimate (SE)	ChiSq	P	Estimate (SE)	ChiSq	P	Estimate (SE)	ChiSq	P
# containers			NS			NS	1.07±0.27	15.42	<0.0001*
Tree 100			NS			NS	-6.40±2.07	9.56	0.0020*
Tree 250			NS			NS	-9.80±3.38	8.41	0.0037*
Urban 100	2.56+1.49	2.93	0.0869	1.91±0.95	4.03	0.0448*			NS
Urban 250	3.86+1.94	3.95	0.0470*	1.97+1.05	3.55	0.0597			NS

**Western transect**	**Elk City**			**Mangum**			**Altus**		
	R^2	DF	P	R^2	DF	P	R^2	DF	P
Sampling Week	0.0556	4	0.0244*	0.2569	4	<0.0001*	0.0962	4	0.0007*
No vegetation			NS	0.0377	1	0.0060*			
Medium vegetation	0.0314	1	0.0092*						
Clutter (not High)			NS	0.0268	1	0.0314*			
Residential sites	0.0252	1	0.0178*						
Dog presence	0.0136	1	0.0685						
Urban (vs Rural)	0.0200	1	0.0476*						
	Estimate (SE)	ChiSq	P	Estimate (SE)	ChiSq	P	Estimate (SE)	ChiSq	P
Tree 100			NS			NS	-2.84+1.20	5.59	0.0181*
Tree 250			NS			NS	-2.82+1.41	4.01	0.0452*

* = significant (Pearson (DF>1/Fisher's Exact (DF=1)/ChiSquare.

Table S**4.** Univariate analysis of *Aedes albopictus* abundance by habitat-related variables by two different traps in 6 cities in Oklahoma between June and August 2017. Results presented are significant (*) or close to significant results for purposes of pattern recognition.

	**Eastern transect**						**Western transect**					
**GAT**	Davis		Ardmore		Marietta		Elk City		Mangum		Altus	
	F Ratio	Prob > F	F Ratio	Prob > F	F Ratio	Prob > F	F Ratio	Prob > F	F Ratio	Prob > F	F Ratio	Prob > F
Sampling Week	3.47	0.013*	7.38	0.0001*	2.28	0.072	1.50	NS	13.75	<0..0001*	13.14	<0.0001*
High vegetation		NS	5.59	0.0221*		NS	4.33	0.0420*		NS		NS
Tree 100		NS	5.23	0.0265*	6.09	0.0165*	3.79	0.0564	3.31	0.0728		NS
Tree 250	3.14	0.0814	3.46	0.0690		NS	5.08	0.0281*		NS		NS
Residential sites	3.04	0.0862		NS		NS	16.39	0.0002*	3.23	0.0456*		NS
Clutter (Y/N)		NS	4.18	0.0463*		NS		NS		NS		NS
Medium clutter	5.56	0.022*		NS	9.58	0.0030*		NS		NS		NS
Dog presence		NS		NS		NS	4.18	0.0455*		NS		NS

**Sentinel**	Davis		Ardmore		Marietta		Elk City		Mangum		Altus	
	F Ratio	Prob > F	F Ratio	Prob > F	F Ratio	Prob > F	F Ratio	Prob > F	F Ratio	Prob > F	F Ratio	Prob > F
Sampling week		NS		NS		NS		NS	3.82	0.0142*		NS
Med vegetation		NS		NS	6.99	0.0170*	6.56	0.0175*		NS		NS
Tree 100		NS	5.98	0.0210*		NS		NS		NS	4.25	0.0533
Tree 250		NS		NS		NS	5.81	0.0244*		NS		NS
# containers		NS		NS	11.83	0.0074*	5.55	0.0349*		NS		NS
Urban 100	6.61	0.0148*	4.82	0.0365*		NS		NS		NS		NS
Urban 250	3.94	0.0555		NS		NS		NS		NS		NS
